# Growth Performance, Blood Biochemical Indices, Rumen Bacterial Community, and Carcass Characteristics in Goats Fed Anthocyanin-Rich Black Cane Silage

**DOI:** 10.3389/fvets.2022.880838

**Published:** 2022-04-25

**Authors:** Ngo Thi Minh Suong, Siwaporn Paengkoum, Jan Thomas Schonewille, Rayudika Aprilia Patindra Purba, Pramote Paengkoum

**Affiliations:** ^1^School of Animal Technology and Innovation, Institute of Agricultural Technology, Suranaree University of Technology, Muang, Thailand; ^2^School of Animal Sciences, Agriculture Department, Can Tho University, Can Tho City, Vietnam; ^3^Program in Agriculture, Faculty of Science and Technology, Nakhon Ratchasima Rajabhat University, Muang, Thailand; ^4^Department of Population Health Sciences, Faculty of Veterinary Medicine, Utrecht University, Utrecht, Netherlands; ^5^Department of Health, Faculty of Vocational Studies, Airlangga University, Surabaya, Indonesia

**Keywords:** anthocyanins, antioxidant capacity, agricultural waste, animal performance, carcass characteristics, rumen

## Abstract

The goal of this study was to investigate the effects of a standard total mixed ration (TMR) with containing anthocyanin-rich plants on animal performance, blood biochemical indices, rumen fermentation, microbial composition, and carcass characteristics in meat goats. Thirty-six healthy crossbred Thai-native Anglo-Nubian male goats (14.42 ± 0.6 kg) were used to compare the possibility of using anthocyanin-rich black cane silage (AS) in place of Napier grass silage (NS) as a functional feed resource. All goats received a 90-d routine feeding consisting of two experimental diets that were isocaloric and isonitrogenous: the control group received TMR containing 50% NS (NS; *n* = 18), and one group received TMR containing 50% AS (AS; *n* = 18). Average daily weight gain (ADG) and dry matter intake (DMI) were measured as indicators of performance. At the end of the experiment, meat, blood, and rumen samples were collected. There were no differences between the two groups in terms of final body weight, ADG, DMI, or ADG/DMI. There were no differences in rumen pH or total volatile fatty acids (VFAs); however, rumen ammonia N concentrations were lower in the AS group than in the NS group. Individual VFA concentrations varied, with AS group containing more *Ruminococcus albus* and NS group containing more methanogenic bacteria. Blood biochemical indices varied, with NS group having higher TBARS concentration and AS group having higher concentrations of TAC, SOD, CAT, GSH-Px, and GSH-Rx. Goat meat from the AS group had higher levels of intramuscular fat and was more tender compared to goat meat from the NS group. The feeding of anthocyanin-rich black cane appears to be an attractive alternative for Napier grass in the nutrition of meat goats. The current results indicate that the feeding of a TMR containing 50% anthocyanin-rich black cane alleviates oxidative stress and promotes the production of tender meat.

## Introduction

Natural phenolic compounds, such as anthocyanin, have gained popularity in recent years due to their health-improving properties, such as their potential as a viable alternative to antibiotics and synthetic growth promoters in the production of sustainable animal feed. Anthocyanin can act as an antioxidant and has been shown to be instrumental in preventing the oxidation of milk and meat (1–3). Thus, the feeding of anthocyanin-rich plants, or byproducts, can be considered opportune in meat goats, especially under tropical conditions. Indeed, heat stress-induced oxidative stress is a well-known constraint for animal performance and health in tropical areas (4, 5).

Anthocyanin-rich black cane (*Saccharum sinensis* Robx.) has recently been recognized as an alternative sugar production technology in Thailand, and the leftover stalks and leaves are potentially of interest in ruminant nutrition. Anthocyanin-rich black cane is a cross between two grasses of the Poaceae family, i.e., *Saccharum spontaneum* and *Saccharum officinarum*, which are endemic to Southeast Asia's mainland (6). The total yield of anthocyanin-rich black cane has been estimated to be 1.2 × 10^7^ metric tons of dry matter (DM) in 2021 from 1.58 × 10^6^ ha in Thailand (7, 8). Thereby, in view of its availability and intrinsic anthocyanin content, anthocyanin-rich black cane can be considered as a valuable feed resource in ruminant nutrition, including meat goats.

To the author's knowledge, no information exists regarding the feeding of anthocyanin-rich black cane to meat goats under practical conditions. It is generally accepted that anthocyanins retain their activity in the rumen environment and thus pass unchanged into the intestine (1). As a result, anthocyanins become available for absorption and can potentially exert their antioxidative effects to protect against oxidative stress. We therefore measured several indices of oxidative stress in blood alongside some general health indicators and hypothesized that feeding anthocyanin-rich black cane reduces the TBARS concentration while increasing the total antioxidant capacity (i.e., TAC) in blood.

There are, however, indications that phenol-rich plants interfere with the microbial community in the rumen fluid (9, 10). Moreover, Tian et al. (11) found that anthocyanin derived from purple corn induced an increase in the acetate to propionate ratio, in combination with a shift in the structure and relative abundance of the microbial population in the rumen of growing goats. These results fuel the idea that anthocyanin-rich black cane may likewise affect rumen fermentation. On the other hand, considerable variation exists in the molecular structure within anthocyanins between plant species (12, 13) which hinders the direct extrapolation of results reported by Tian et al. (11). Thus, we considered it opportune to investigate the effect of anthocyanin-rich black cane on selected rumen indices, thereby contributing to the generalization of the current knowledge on anthocyanins in ruminant nutrition. Moreover, an anthocyanin induced increase in ruminal acetic acid production potentially renders greater amounts of substrate available for *de-novo* fat synthesis (14). We therefore hypothesize also that the feeding of anthocyanin-rich black cane increases the fat content of goat meat.

## Materials and Methods

### Silage Preparation

Napier grass and anthocyanin-rich black cane were grown and harvested at the goat and sheep research farm of Suranaree University of Technology (SUT), Nakhon Ratchasima, Thailand (14°52'49.1“N, 102°00'14.9”E, 243 m above sea level). The two grasses were grown between August 2017 and January 2018, during the monsoon season. After harvesting, the Napier grass and anthocyanin-rich black cane materials were chopped (2–3 cm using a crop cutter) and ensiled without wilting (8, 15). Both silages fermented well after 90 d of ensiling, as indicated by sensory evaluation and a pH value measured using a portable pH meter (Eutech Pc 700, Italia) when the drum containing the silage was opened. The chemical composition and fermentation characteristics of Napier grass silage (NS) and anthocyanin-rich black cane (AS) are provided in Supplementary Table S1.

### Experimental Design

Thirty-six healthy crossbred Thai-native Anglo-Nubian male goats with a body weight (BW) of 14.42 kg (± 0.6 SD) were selected among 500 goats reared by all collaborating farmers in Thailand. The goats were divided into two groups, housed individually, and randomly assigned to one of two experimental diets (Table 1): (1) Napier grass silage-based diet (a diet containing 50% NS, *n* = 18), or (2) anthocyanin-rich black cane silage-based diet (a diet containing 50% AS, *n* = 18). The remaining ingredients were identical to those used in a basal total mixed ration (TMR) diet prepared in accordance with the NRC requirements (16). An adjustment period of 14 d allowed the goats to become acclimated to routine feeding and to allow time for proper diet adjustment prior to the experiment. During the adjustment period, the animals were gradually fed experimental diets. Following the 14-d period, the animals were fed the experimental diets on a daily basis for 90 d. The animals were offered on average ~350 g DM of TMR/day, which was supplied in two equal portions at 0700 and 1,600. Throughout the experiment, the goats had free access to fresh water and a trace mineral salt block. On average, the animals consumed ~95% of the amount of feed supplied. Daily feed refusals were recorded and used to calculate DM intake (DMI). The BW of the goats was recorded to determine their growth performance throughout the routine feeding.

**Table 1:** Ingredients and nutrient composition of Napier grass silage (NS) and anthocyanin-rich black cane (AS).

**Item**	**Experimental diet**	
	**NS**	**AS**
Ingredient, DM basis		
Napier grass silage	50.0	0.0
Anthocyanin-rich black cane silage	0.0	50.0
Cassava pulp	3.8	3.8
Cassava chip	19.5	19.5
Mineral mix^a^	0.8	0.8
Palm meal	14.0	14.0
Premix^b^	0.2	0.2
Rice bran	5.5	5.5
Soybean meal	4.0	4.0
Sulfur^c^	0.4	0.4
Sunflower oil	1.0	1.0
Urea	0.9	0.9
Nutrient composition, DM basis		
Metabolizable energy, Mcal/kg	17.5	17.3
Crude protein, %	11.7	11.6
Neutral detergent fiber, %	53.6	52.9
Acid detergent fiber, %	24.5	24.4
Acid detergent lignin, %	3.8	3.4
Hemicellulose, %	29.1	28.5
Cellulose, %	20.8	21.0
Ash, %	10.6	10.5
Total anthocyanins, mg/g	0.03	0.17
Anthocyanin profile, % of total anthocyanins		
Cyanidin-3-glucoside	12.86	4.83
Pelargonidin-3-glucoside	22.30	7.24
Delphinidin	17.12	16.90
Peonidin-3-O-glucoside	8.66	16.21
Malvidin-3-O-glucoside	2.46	14.14
Cyanidin	4.03	25.17
Pelargonidin	4.03	1.72
Malvidin	28.52	13.79

a *Contained (g/kg): NaCl (600), P (160), Ca (240)*.

b *Vitamin A (4,200.000 IU/kg), vitamin A_3_ (840,000 IU/kg), vitamin E (10,000 IU/kg), vitamin K_3_ (2 g/kg), vitamin B_1_ (2.4 g/kg), vitamin B_2_ (3.5 g/kg), vitamin B_6_ (1.8 g/kg), vitamin B_12_ (0.01 g/kg), vitamin B_5_ (4.6 g/kg), vitamin C (12 g/kg), folic acid (0.28 g/kg), vitamin 7 (0.4 g/kg), coper (12 g/kg), manganese (40 g/kg), zinc (3.2 g/kg), iron (42 g/kg), iodine (0.8 g/kg), cobalt (0.8 g/kg), selenium (0.35 g/kg)*.

c *Sulfur cube was derived from commercial purchase (Sand Sea Sun Shop: TG-6731, Bangkok, Thailand) and ground (sieve size of 1 mm)*.

### Sample Collection and Laboratory Analysis

Samples of dietary ingredients, two experimental diets, and feed refusals were collected every 2 wk, dried at 55°C in an air oven, and ground in a Wiley Mill using (Retsch SM 100 mill; Retsch Gmbh, Haan, Germany) through a 1-mm screen. The dried samples were stored for chemical and nutrient content analysis. The contents of neutral detergent fiber (NDF) (with heat stable α-amylase), acid detergent fiber (ADF), and acid detergent lignin (ADL) were determined according to Van Soest et al. (17), with the use of a fully automated system (FibertecTM 8000, FOSS, Hilleroed, Denmark). The hemicellulose content of the feed was calculated as NDF minus ADF, whereas the cellulose content of the feed was calculated as ADF minus ADL. The nitrogen content of two experimental diets and diet refusals were analyzed with a Kjeltec™ 8400 fully automated Kjeldahl analyser (FOSS, Hilleroed, Denmark); 6.25 was used as the conversion factor to obtain crude protein (CP) values. Furthermore, the second subsample was extracted at 50°C for 24 h with 0.01 N hydrochloric acid (HCl) dissolved in an 80% methanol solution, and the supernatant was collected and transferred into a 50-ml volumetric flask for HPLC determination of anthocyanin composition (15, 18, 19). The chromatographic separation was carried out on a reversed-phase Zorbax SB-C18 column (3.5 μm particle size, i.d. 4.6 mm × 250 mm, Agilent Technologies, Santa Clara, CA, USA) for 65 min at 28°C. The injection volume was kept constant at 20 μl. The mobile phase was composed of acetonitrile of HPLC grade and 10% acetic acid (1:9). Chromatographic separation was accomplished at a flow rate of 0.8 ml/min using a binary gradient of (A) 10% acetic acid, 5% acetonitrile, and 1% phosphoric acid, and (B) acetonitrile. The absorption of the compounds that were tested and measured was evaluated using a photodiode array UV detector set to 520 nm. The Agilent OpenLAB CDS 1.8.1 system manager was used to make sure that the data was correct and to analyze chromatographic data. When there were differences in the nutritional values of the diet between the measured and initial values, the dietary formula was adjusted according to the measured nutrient values.

Blood samples were taken 2 h after morning feeding at 09:00 on the last feeding wk through jugular venipuncture into a single 10-ml heparin-containing vacuum tube. The samples were placed on ice for transfer to the laboratory. Subsamples (5 ml) were centrifuged at 5,000 × g for 20 min at 4°C (Sorvall Legend XT/XF Centrifuge Series, Thermo Fisher Scientific, Waltham, MA, USA), and collected plasma were frozen at −20°C until analysis. Plasma concentrations of urea, total protein, glucose, insulin, triglycerides, alanine transaminase, and aspartate aminotransferase were determined using an automated enzymatic colorimetric method on a Cobas Integra 400 Instrument (Roche Diagnostics, Mannheim, Germany), quadruplicate, as described by Xue et al. (20) and Niu et al. (21). Other subsamples were centrifugated at 3,000 × g for 15 min at 4°C. Plasma was then used to determine plasma antioxidant (TAC, TBARS, SOD, CAT, GSH-Px and GSH-Rx) using an automated enzymatic colorimetric method on a Microplate (96 wells, UV plate), quadruplicate, equipped into microreader (Varioskan-LUX multimode microplate reader, Thermo Scientific, USA) as explained in prior investigation (5).

All goats (*n* = 36) were killed at the end of the experiment. Then, samples (~500 ml, mixture of liquid and solid) were collected from the dorsal, central, and ventral regions of the rumen to form a composite sample, and then strained through four layers of cheesecloth to collect rumen fluid. Then, the pH of the rumen fluid was immediately determined using a handheld pH meter (Eutech Pc 700, Italia). The strained rumen fluid was subsequently put in a sterilized thermos flask and directly transported to the laboratory. Upon arrival at the laboratory, the filtered rumen fluid was divided in two aliquots. The first aliquot of the filtrates (5 ml) was treated with 0.5 ml of 50% (v/v) HCl, 0.5 ml of a metaphosphoric acid solution (187.5 g/L) and a formic acid (250 ml) solution and stored at −18°C pending the chemical analysis of ammonia-N and volatile fatty acid (VFA) concentrations. After thawing, the concentration of ammonia N in the filtrates was determined using a micro-Kjeldahl method [Kjeltec 8100, Hillerd, Denmark, AOAC (22, 23)]. The concentration of VFAs in the filtrates was quantified using a gas chromatography (Agilent 6890 GC, Agilent Technologies, Wilmington, DE, USA) with a 30 m × 0.25 mm × 0.25 μm column (DB-FFAP) and peak detection was compared and calculated as described by Purba et al. (24). The analyses for ammonia N and VFAs were carried out in quadruplicate, and the mean data was used for statistical analysis.

The second aliquot of the filtrates (5 ml) was homogenized for microbiological detection and stored at −80°C until the analysis the relative abundances of selected rumen microbes. After thawing, DNA was extracted from the homogenized filtrates by means of a quantitative real-time PCR (qPCR) reaction. Total genomic DNA was extracted from 1 ml homogenized filtrates using the QIAamp DNA Stool Mini Kit (Qiagen, Hilden, Germany) following the RBB+C method (25). The DNA yield was determined using a NanoDrop NanoVue spectrophotometer (GE Healthcare Bio-Sciences, Pittsburgh, PA, USA) set to a 260:280 absorbance ratio. To prevent the DNA from degradation, the DNA yield was eluted with appropriate dilutions (volume of nuclease-free water) and stored at −20°C until further analysis. The relative abundances of selected primers in genomic DNA extracted from rumen fluids were quantified using a QuantiTect SYBR Green RT–PCR Kit (full master mix; Qiagen) fitted with the selected primer set and a Roche Lightcycler 480-II (Roche Applied Science, Basel, Switzerland), following the recently reported amplification and qPCR settings (26). References for the relative abundances of total bacteria, *Ruminococcus albus, Ruminococcus flavefaciens, Fibrobacter succinogens, Butyrivibrio fibrisolvens, Megasphaera elsdenii, Streptococus bovis*, Methanogen, and Protozoa were obtained from Vivantis Technologies Sdn Bhd (Selangor Darul Ehsan, Malaysia). The primers used for qPCR were forward primer 5′-CGGCAACGAGCGCAACCC-3′ and reverse primer 5′-CCATTGTAGCACGTGTGTAGCC-3′, forward primer 5′-TCTGGAAACGGATGGTA-3′ and reverse primer 5′-CCTTTAAGACAGGAGTTTACAA-3′, forward primer 5′-GTTCGGAATTACTGGGCGTAAA-3′ and reverse primer 5′-CGCCTGCCCCTGAACTATC-3′, forward primer 5′-TTCGGTGGATCDCARAGRGC-3′ and reverse primer 5′-GBARGTCGWAWCCGTAGAATC-3′, and forward primer 5′-CTTGCCCCTCYAATCGTWCT-3′ and reverse primer 5′-GCTTTCGWTGGTAGTGTATT-3′, for total bacteria, *R. flavefaciens, F. succinogens*, methanogen, and protozoa, respectively (27); forward primer 5′-ACACACCGCCCGTCACA-3′ and reverse primer 5′-TCCTTACGGTTGGGTCACAGA-3′, forward primer 5′-GACCGAAACTGCGATGCTAGA-3′ and reverse primer 5′-CGCCTCAGCGTCAGTTGTC-3′, for *B. fibrisolvens* and *M. elsdenii*, respectively (28); forward primer 5′-CCCTAA AAGCAG TCTTAGTTCG-3′ and reverse primer 5′-CCTCCTTGCGGTTAGAACA-3′ for *R. albus* (29); forward primer 5′-GCCA GGCTATTTAGGTGACACTATAG-3′ and reverse primer 5′-GGGT AATACGACTCACTATAGGG-3′, for *S. bovis* (30). Prior to beginning qPCR tests, a standard curve was established using a 6 fold serial dilution of pooled DNA. To ensure reproducibility, qPCR tests were performed in quadruplicate for each selected species or group of bacteria using both standards and genomic DNA samples. The Ct data were transformed into normalized relative numbers using the LightCycler 480 software version 1.2.9.11 (Roche Applied Science, Basel, Switzerland), which accounted for PCR efficiency. The values for the 16S rRNA gene of a particular species or group of microorganisms are defined as a relative percentage of total bacteria.

### Goat Slaughter and Carcass Measurements

At the culmination of the routine feeding trail, goats were fasted for 24 h before being loaded (at 0500 h) and transported half a mile to a university slaughterhouse. Goats were spared as much as possible. Briefly, large ramps with steep slopes (11°) were employed for the loading and unloading of goats, and non-slip flooring was installed on the floors of the transport vehicles. Once the transport vehicles arrived at the slaughterhouse, effective scheduling processes were developed to guarantee rapid unloading of goats. To assist the smooth movement of goats, pre-slaughter handling methods were deployed. The goats were slaughtered standing up, with their heads fastened and their necks exposed for the throat incision. The goat knifes had a long, razor-sharp, and unbroken blade. These appropriate knives were prepared to cause a rapid outpouring of blood by cutting both jugular veins and both carotid arteries.

Prior to slaughter, the final BW of each goat was determined to establish the dressing percentage. The hot carcass weight was determined on the day of slaughter, and the carcasses were then refrigerated at 4°C for 48 h after slaughter. The weights of cold carcasses and the weights of shrinking carcasses were determined. Between the 12^th^ and 13^th^ ribs, carcasses were cut up to evaluate carcass quality features and yield grade metrics. Carcass quality characteristics included 12^th^-rib fat thickness, longissimus muscle (LM) area, intramuscular fat, and final pH values. Carcass color was determined by measuring the L^*^, a^*^, and b^*^ color values on the cut lean surface and carcass external fat along the lateral side of the carcass with a portable Chroma Meter (CR-300, Minolta Corporation, Osaka, Japan). Quadruplicate color readings were done in the L^*^ (0 = black, 100 = white), a^*^ (negative values = green, positive values = red), and b^*^ (negative values = blue, positive values = yellow) color space (CIELAB); big chunks of connective tissue and intramuscular fat were excluded. Color saturation was estimated in accordance with the operational manual (31).

### Meat Measurements

The Warner-Bratzler shear force (WBSF) values were calculated using the AMSA (1995) recommendations with slight modifications (32). Following the collection of carcass data, strip loins were removed from the left side of each carcass, vacuum-packed, and stored for 14 d at 4°C. Following the aging period, 2.54-cm-thick steaks were prepared, vacuum bagged, and frozen (−20°C) for further examination. WBSF steaks were defrosted at 4°C for 24 h and grilled to an internal temperature of 70°C on an electric grill (Kashiwa KW-308, Nakhon Pathom, Thailand) equipped with thermocouples inserted about in the geometric center of the steak. Cooking loss, reported as a percentage of weight loss, was obtained by dividing the initial weight (before cooking) by the end weight (after cooking). Using a steel hollow-core equipment, at least six 1.27-cm-diameter pieces were excised from each steak parallel to the muscle fiber orientation (32). The pieces were sheared perpendicular to the muscle fiber orientation using a shear device Texture analyzer for Warner-Bratzler Meat Shear (TA-TX2 Texture Analyzer, Stable Micro Systems, UK). A crosshead speed of 200 mm/min was used, as well as a 5 kN load cell calibrated to 113 read over the range 0x100 N. Six pieces were evaluated for their peak shear forces and expressed in kilograms.

External fat and connective tissue were removed from meat samples to determine ash, protein, moisture, and ether extractable lipid. In brief, CP content was estimated from nitrogen content (%N × 6.25) using a KjeltecTM 8400 fully automated Kjeldahl analyser (FOSS, Hilleroed, Denmark); ash content was determined by heating the steak sample using a furnaces (Carbolite AF1100, Parsons Lane, England) at 550°C for 15 h; moisture content was evaluated by weight loss after freeze-drying at −55°C for 5 d; and lipid content was determined using a Soxtec 2050 [Foss, Höganäs, Sweden, AOAC (22)]; petroleum ether 35–60°C used for extractable solvent. The analyses were carried out in quadruplicate, and the mean data was used for statistical analysis.

### Statistical Analysis

Data on animal performance, blood biochemical indices, rumen fermentation, microbial community, and carcass characteristics were statistically analyzed using the SAS 9.4 general linear model procedure (33) using the model: Y_ij_ = μ+ τ_i_ + ε_ij_, where Y_ij_ is the response variable, μ is the overall mean, τ_i_ is experimental diet (*i* = NS or AS), and ε_ij_ is the residual error. In this model, diet was considered as a fixed effect, while the animal was considered as a random effect. The Shapiro–Wilk test was used to normalize all data. All of the data was analyzed using the Student's *t*-test. The least-squares means were reported, and *P* <0.05 was declared significant.

## Results

### Experimental Diets, Feed Intake, and Growth Performance

The chemical composition of the experimental diets is shown in Table 1. The experimental diets were isocaloric and isonitrogenous. Moreover, the contents of hemicellulose, cellulose, and lignin were similar between the two diets. The anthocyanin content of the AS *vs*. NS was found to be almost 6 times higher. Within the anthocyanin fraction, the differences between AS and NS were most pronounced for the Malvidin-3-O-glucoside and Cyanidin fractions, i.e., 5.7 and 6.2 times higher in AS compared to NS (Table 1).

The DMI, ADG, and the ratio between ADG and DMI of the crossbred Thai-native Anglo-Nubian male goats are summarized in Table 2. The AS diet had the same effect on animal growth performance as the NS it replaced (*P* > 0.05) after 90 d of routine feeding.

**Table 2:** Growth performance of goats fed a total mixed ration supplemented with Napier grass silage (NS) or anthocyanin-rich black cane silage (AS).

**Item^**a**^**	**Experimental diet**		**SEM**	***P*** **-value**
	**NS**	**AS**		
Animal number	18	18		
Initial body weight, kg	14.38	14.42	0.251	0.208
Final body weight, kg	17.25	17.65	0.321	0.135
ADG, g/d	34.13	38.49	0.542	0.289
DMI, g/d	362.87	334.07	0.313	0.427
ADG/DMI	0.09	0.12	0.404	0.177

a *ADG, average daily weight gain; DMI, dry matter intake; SEM, standard error of mean*.

### Rumen Fermentation and Microbial Community

The variables of rumen fermentation are summarized in Table 3. The pH values of rumen fluids were similar between the two groups. The rumen ammonia N concentration was found to be 5.2% lower (*P* = 0.002) when the goats were fed AS. In contrast, the total VFA concentration was ~ 6.8% higher (*P* = 0.001) when AS was fed to the goats. The feeding of AS, instead of NS, caused higher proportions of acetate (*P* = 0.005) and lower proportions of propionate (*P* = 0.026). Consequently, the acetate to propionate ratio was found to be 13.5% higher (*P* = 0.003) after the feeding of AS. The proportions of butyrate were not affected by the feeding of AS (*P* = 0.401).

**Table 3:** Rumen fermentation of goats fed a total mixed ration supplemented with Napier grass silage (NS) or anthocyanin-rich black cane silage (AS).

**Item^**a**^**	**Experimental diet**		**SEM**	***P***-**value**
	**NS**	**AS**		
pH value	6.74	6.78	0.123	0.348
Ammonia N, mg/dl	14.11	13.37	0.343	0.002
Total VFAs, mM	79.07	84.42	0.420	0.001
Individual VFA, molar % of total VFAs			
Acetate	57.43	59.35	0.654	0.005
Propionate	30.96	28.30	0.389	0.026
Butyrate	11.61	12.35	0.684	0.401
Acetate/Propionate	1.85	2.10	0.088	0.003

a *VFAs, volatile fatty acids; SEM, standard error of mean*.

**Figure 1:**
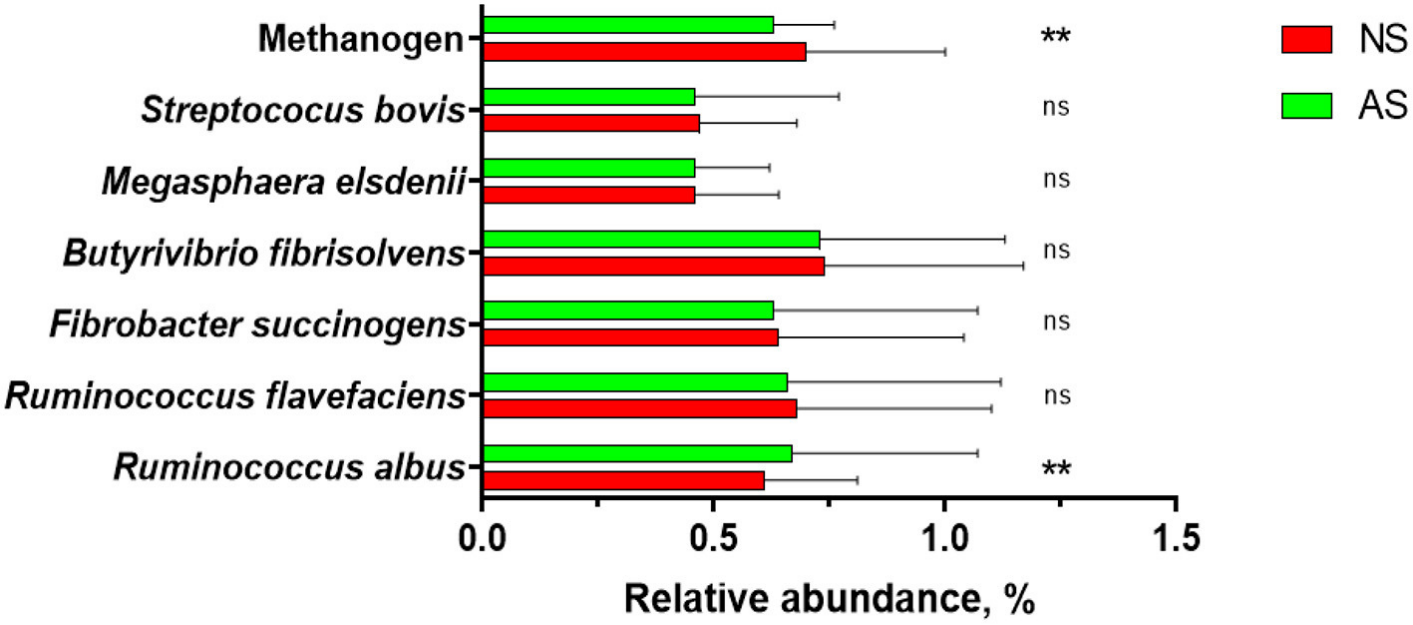
The relative abundances of selected microbial rumen of goats fed a total mixed ration supplemented with Napier grass silage (NS) or anthocyanin-rich black cane silage (AS). Values are means, with their standard errors represented by horizontal bars. Values was different (**P < 0.001); ^ns^*P* > 0.05).

Both, the abundances of total bacteria and total protozoa (*P* = 0.432) in rumen fluid of the goats were similar between the two diets, i.e., *P* = 0.619 and *P* = 0.432, respectively. Overall, total protozoa and total bacteria of two groups were found to have log_10_ copies of 16 rRNA genes ranging from 6.22 to 6.31 and 9.35 to 9.46, respectively. The relative abundances of *R. flavefaciens, F. succinogens, B. fibrisolvens, M. elsdenii*, and *S. bovis* (Figure 1), expressed as percentage of the 16S rRNA gene copy number of the total bacteria (%), were similar between the two groups (*P* ≥ 0.118). The relative abundance of methanogen was found to be lower (*P* = 0.001) when AS was fed while the relative abundance of *R. albus* was found to be higher (*P* = 0.007) after the feeding of AS.

### Blood Biochemical Indices

The AS diet did not affect (*P* ≥ 0.110) on plasma concentrations of glucose, albumin, cholesterol, insulin, triglycerides, HDL, LDL, VLDL, or plasma urea nitrogen (Table 4). Similarly, there were no significant differences in the concentrations of IgG, alanine transaminase or aspartate aminotransferase between the two groups (*P* ≥ 0.324). The concentration of TBARS, however, was almost 9% lower (*P* = 0.024) when AS was fed to the goats (Table 4). In contrast, the feeding of AS instead of NS caused higher plasma concentrations of TAC (*P* = 0.002) and also higher activities of SOD, CAT, GSH-Px, and GSH-Rx (*P* ≤ 0.009).

**Table 4:** Blood biochemical indices of goats fed a total mixed ration supplemented with Napier grass silage (NS) or anthocyanin-rich black cane silage (AS).

**Item^**a**^**	**Experimental diet**		**SEM**	***P*** **-value**
	**NS**	**AS**		
Total protein, g/L	73.10	72.40	0.861	0.312
Albumin, g/L	33.63	33.30	0.872	0.244
Globulin, g/L	39.47	39.10	0.684	0.218
Blood urea N, mmol/L	8.03	8.00	0.150	0.110
Insulin, μU/ml	1.64	1.72	0.338	0.591
Glucose, mmol/L	4.93	5.12	0.429	0.372
Total cholesterol, mmol/L	3.41	3.12	0.877	0.284
Triglyceride, mmol/L	0.61	0.57	0.085	0.259
HDL, mmol/L	1.87	1.72	0.336	0.146
LDL, mmol/L	0.94	1.02	0.430	0.385
VLDL, mmol/L	1.21	1.14	0.458	0.138
Alanine transaminase, U/L	29.85	29.74	0.660	0.324
Aspartate aminotransferase, U/L	34.96	35.05	0.625	0.560
IgG, g/L	12.01	12.13	0.520	0.450
TAC, nmol/μl	25.48	32.62	0.621	0.002
SOD, U/ml	74.65	83.75	0.451	0.009
CAT, U/ml	60.79	68.20	0.627	0.010
GSH-Px, U/ml	39.54	43.98	0.323	0.028
GSH-Rx, U/ml	42.76	53.18	0.377	0.011
TBARS, nmol/ml	44.06	40.15	0.443	0.024

a *HDL, high density lipoprotein; LDL, low density lipoprotein; VLDL, very low-density lipoprotein; IgG, immunoglobin G; TAC, total antioxidant capacity; SOD, superoxide dismutase; CAT, catalase; GSH-Px, glutathione peroxidase; GSH-Rx, glutathione reductase; TBARS, thiobarbituric acid-reactive substances; SEM, standard error of mean*.

### Carcass and Meat Characteristics

Both the hot and cold carcass weights at slaughter (Table 5) were similar between the two diets (*P* ≥ 0.194). Consequently, the dressing percentage also was similar between diets (*P* = 0.217). Likewise, the 12^th^ rib fat thickness, LMA, and pH were not affected by AS (*P* ≥ 0.157). There were no significant differences between the two groups in the indices related to the external fat color or 12^th^ rib lean color (*P* ≥ 0.235).

**Table 5:** Carcass characteristics of goats fed a total mixed ration supplemented with Napier grass silage (NS) or anthocyanin-rich black cane silage (AS).

**Item^**a**^**	**Experimental diet**		**SEM**	***P*** **-value**
	**NS**	**AS**		
BW, kg	17.25	17.65	0.321	0.135
HCW, kg	8.15	8.50	0.634	0.194
CCW, kg	8.05	8.48	0.839	0.294
DP, %	47.25	48.16	0.675	0.217
12^th^-rib fat thickness, cm	0.24	0.23	0.033	0.246
LMA, cm^2^	14.53	14.50	0.642	0.157
pH value	6.87	6.84	0.532	0.214
External fat color^b^, ^c^				
L^*^	21.48	21.90	0.639	0.589
a^*^	16.45	15.07	0.306	0.235
b^*^	6.35	6.58	0.409	0.440
c^*^	17.63	16.44	0.324	0.391
12^th^-rib lean color				
L^*^	46.70	48.68	0.694	0.432
a^*^	10.50	9.84	0.440	0.266
b^*^	5.92	6.70	0.367	0.363
c^*^	12.05	11.90	0.442	0.329

a *BW, body weight; HCW, hot carcass weight; CCW, cold carcass weight; DP, dressing percentage; LMA, LM area measured at 12^th^ rib; SEM, standard error of mean*.

b *Fat color measurements obtained approximately 20 cm ventrally to the lateral process of the split carcass adjacent the 13^th^ rib*.

c CIE color measurements: L^*^ = lightness, black (0) to white (100); positive a^*^ =red; negative a^*^ = green; positive b^*^ = yellow; negative b^*^ = blue; c^*^ = color saturation = [(a^*^)^2^ + (b^*^)^2^]^1/2^ whereby a large number is considered to be more vivid.

As shown in Table 6, the cooked rate, drip loss, moisture, protein, and ash percentages of steaks were similar between the two experimental diets (*P* ≥ 0.111). However, the WBSF value of tenderness was ~ 13.8% lower (*P* = 0.035) when AS was fed. In contrast, the values on the percentage of intramuscular fat in steak samples were found to be ~ 28% higher (*P* = 0.015) after the feeding of AS.

**Table 6:** WBSF and chemical composition of steak samples of goats fed a total mixed ration supplemented with Napier grass silage (NS) or anthocyanin-rich black cane silage (AS).

**Item^**a**^**	**Experimental diet**		**SEM**	***P*** **-value**
	**NS**	**AS**		
WBSF, kg	8.58	7.41	0.191	0.035
Cooked rate, %	43.17	42.96	0.336	0.266
Drip loss, %	6.54	8.66	0.493	0.111
Moisture, %	78.76	79.25	0.322	0.422
Protein, % DM	88.57	88.35	0.315	0.648
Intramuscular fat, % DM	4.44	5.67	0.165	0.015
Ash, % DM	7.16	6.17	0.277	0.704

a *WBSF, Warner-Bratzler shear force values of tenderness; SEM, standard error of mean*.

## Discussion

To the best of the author's knowledge, the current *in-vivo* results are the first reporting on feeding of anthocyanin-rich black cane and the data indicate that the feeding of anthocyanin-rich black cane, instead of Napier grass, caused a greater relative abundance of *R. albus*. On the other hand, the relative abundances of *R. flavefaciens, F. succinogens, B. fibrisolvens, M. elsdenii*, and *S. bovis* were not affected by AS. Thus, the overall impact of AS on the selected rumen microbes can be considered minor. This observation seems to be in line with results reported by Tian et al. (11). Indeed, the latter reported that supplemental purple corn anthocyanin (PCA) had no effect on most rumen bacteria with a relative abundance > 1% at the genus level. In the current study, the total anthocyanin content of diet was ~ 0.02% (DM basis) while in the study of Tian et al. (11), a maximum PCA content of ~ 0.14% (DM basis) was used. It can therefore be speculated that the total anthocyanin content of the AS-diet was too low to effectively influence the microbial population The feeding of AS induced a reduction in the relative abundance of the methanogens. This result is in line with observations in sheep (34) showing a reduced relative abundance of the methanogens in rumen fluids after the feeding of mulberry leaf. The reduction in the relative abundance of the methanogens can be interpreted in that the feeding of AS reduces methane production. This interpretation is, however, not in line with the observed increase in the rumen acetate to propionate ratio. The latter observation can be interpretated in that the feeding of AS promotes the production of methane. Indeed, it is generally accepted that the production of acetate, instead of propionate, provides a source of hydrogen in the synthesis of acetate (35–37). Clearly the, at least apparent, discrepancy in the current results on acetic acid and relative abundance of methanogens is not easy to explain but it cannot be excluded that hydrogen was used by acetogenic bacteria to synthesize acetate. Indeed, acetogenic bacteria are able to convert carbon dioxide and hydrogen directly into acetate (38, 39). Unfortunately, neither methane production nor the abundance of acetogenic bacteria were measured in the current *in-vivo* study and the current results do not provide further clues to further speculate on the fate of the hydrogen. Thus, future studies are warranted to shed further light on this issue.

The feeding of anthocyanin-rich black cane, instead of Napier grass, increased the concentration of total VFAs. The latter result can be interpreted as meaning that the AS diet rendered greater amounts of substrate available for fermentation. Our observation of an increased relative abundance of *R. albus* appeared to confirm this notion. These results are corroborated by Yusuf et al. (9) who demonstrated that including *Andrographis paniculata* leaves rich in plant active substances (lactones, anthocyanin, flavonoids, and sterols) in the diet of goats increased the quantity of ruminal *R. albus* while maintaining the total bacteria in the ruminal fluid, thereby improving the digestibility of nutrients. The aforementioned results implicate that anthocyanin-rich black cane may have a higher rumen digestibility than Napier grass. The cellulose, hemicellulose, and CP contents of the two experiments were comparable, as was DMI. The notion that AS supplied greater amounts of carbohydrates to the ruminal bacteria agrees with the observed lower ammonia-N concentrations when AS was fed. It is well established that fermentable carbohydrates play a critical role in stimulating rumen microbe growth and thus in converting rumen degradable CP to microbial protein (40, 41). Alternatively, it cannot be excluded that the increase in anthocyanin intake decreased the rumen solubility of the dietary proteins and therefore lowered the rumen ammonia N concentrations (11). Clearly, the current study provides no additional information on anthocyanin-induced protein solubility reduction, and further research is needed to resolve this issue. Finally, the increased total VFA concentrations caused by the AS diet were not associated with increased DMI or improved meat goat growth performance. Thus, the relevancy of the greater total VFA concentrations can be disputed. Unfortunately, neither the fermentable organic matter nor the apparent fecal digestibility of the two diets were measured in the current experiment, thereby hindering any substantiation of the notion of rumen digestibility of the two experimental diets.

The current results confirm our hypothesis that the feeding of AS reduced the oxidative stress of the goats, as indicated by the lower TBARS values and greater TAC levels. These results are in line with previous studies reported by Tian et al. (3) and Purba et al. (5). These observations can be explained by the anthocyanins in the AS diet, including cyanidin-3-glucoside, pelargonidin-3-glucoside, delphinidin, peonidin-3-O-glucoside, malvidin-3-O-glucoside, cyanidin, pelargonidin, and malvidin. These anthocyanins have been shown to have anti-oxidative and anti-inflammatory properties (3). However, feeding AS increased antioxidant enzyme activity such as SOD, CAT, and GSH. These findings corroborate those of Purba et al. (5), who reported modulated oxidative indicators such as SOD, CAT, and GSH activities in ruminal fluid, blood, milk, and mammary tissue following feeding of a total mixed ration containing piper meal (*Piper betle* L.) containing flavonoids (possibly including anthocyanin fraction), essential oils, and phenolic acids to lactating goats. Nonetheless, increasing antioxidant enzyme activity in ruminal fluid or blood may result in a decrease in TBARS values as a result of optimizing the rumen and its rumen bilayers for dietary bioactive chemicals derived from piper meal by improving membrane fluidity (10). In the current study, we postulated that increasing anthocyanin consumption in the AS diet could function as an electron donor, neutralizing the accumulation of reactive oxygen species. SOD is the first line of defense against scavenging reactive oxygen species among intracellular enzymes. However, activation of CAT and GSH-Px or GSH-Rx implied that the superoxide anion radical in the dismutation exceeded the limit, resulting in reactive oxygen species being scavenged by CAT or GSH enzymatically before being converted to water (5, 42). SOD, CAT, or GSH all appeared to cooperate in suppressing the scavenging reactive oxygen species. It thus appears that the lower TBARS values and greater TAC levels are not only caused by the antioxidative action of anthocyanin itself, but by also the higher SOD, CAT, and GSH-Px or GSH-Rx expressions.

The feeding of AS did not affect any of the selected carcass characteristics and this result is consistent with previous results in small ruminants (2, 43) while others indicate a favorable effect (21, 44). The variable outcome between studies is likely attributable to the wide variety of factors, including the composition of the anthocyanin-based diet used in the study, the age of slaughter, and the bioavailability of flavonoids or anthocyanins in ruminants. Furthermore, neither the adipose nor the lean tissue color (L^*^, a^*^, b^*^, or c^*^) of goats was changed by anthocyanin-rich black cane, thereby suggesting that lipid peroxidation was similar between the two experimental diets. A reduced meat color (a^*^ and b^*^) tends to brown the meat, suggesting that the oxymyoglobin to metmyoglobin conversion stage and the lipid peroxidation interaction were involved in meat discoloration (43). Anthocyanins containing cyanidin-3-glucoside and malvidin-3-O-glucoside stabilized muscle membranes, resulting in an enhancement in meat color (45, 46). As we found, both the NS and AS diets include cyanidin-3-glucoside and malvidin-3-O-glucoside, which may reduce lipid peroxidation and preserve the meat coloration. Similar findings were obtained following the addition of anthocyanin compounds to the diets of goats (43) and cattle (44).

In the current study, the AS diet resulted in greater intramuscular fat contents. The greater intramuscular fat contents are most likely explained by the greater rumen acetate production when the goats were fed AS. Indeed, a greater production of acetate renders more substrate available for *de-novo* fat synthesis (14). Previous research established a negative correlation between intramuscular fat contents and WBSF tenderness values (47). Thus, it is suggested that the lower WBSF values are, at least partly, explained by the higher intramuscular fat contents. The AS induced lower WBSF value is in line with results reported by Tian et al. (43).

## Conclusions

The feeding of anthocyanin-rich black cane appears to be an attractive alternative for Napier grass in the nutrition of meat goats. The current results indicate that the feeding of a TMR containing 50% anthocyanin-rich black cane alleviates oxidative stress and promotes the production of tender meat. Moreover, the concentration of total volatile fatty acids, in combination with a higher proportion of acetate, was found to be higher when goats were fed anthocyanin-rich black cane instead of Napier grass. The latter may explain both the higher fat content and tenderness of the meat when anthocyanin-rich black cane is fed. Future studies, however, are warranted to confirm the current results and to investigate the effects of dietary anthocyanin-rich black cane on digestion and carcass characteristics including the antioxidant profile, and texture of the meat.

## Data Availability Statement

The original contributions presented in the study are included in the article/Supplementary Material, further inquiries can be directed to the corresponding authors.

## Ethics Statement

The animal study was reviewed and approved by the Animal Ethics Committee of Suranaree University of Technology issued a statement approving the experimental protocol (SUT 4/2558). The research was carried out in accordance with regulations on animal experimentation and the Guidelines for the Use of Animals in Research as recommended by the National Research Council of Thailand (U1-02632-2559). Written informed consent for participation was not obtained from the owners because we used our goat groups and those includes in ethical considerations.

## Author Contributions

NS: conceptualization, methodology, formal analysis, investigation, resources, data curation, writing—review and editing, visualization, and project administration. SP: conceptualization, methodology, writing—review and editing, supervision, project administration, and funding acquisition. JS: conceptualization, methodology, data curation, and writing—review and editing. RP: conceptualization, methodology, formal analysis, investigation, resources, data curation, writing—original draft preparation, writing—review and editing, visualization, supervision, project administration, and funding acquisition. PP: conceptualization, methodology, resources, data curation, writing—review and editing, supervision, project administration, and funding acquisition. All authors contributed to the article and approved the submitted version.

## Funding

This research was funded by (i) Suranaree University of Technology (SUT; contract no. Full-time 61/02/2021), (ii) Thailand Science Research and Innovation (TSRI), (iii) National Science, Research and Innovation Fund (NSRF; project codes: 90464; 160368), (iv) National Research Council of Thailand (NRCT; project code: 900105), and (v) Nakhon Ratchasima Rajabhat University (NRRU).

## Conflict of Interest

The authors declare that the research was conducted in the absence of any commercial or financial relationships that could be construed as a potential conflict of interest.

## Publisher's Note

All claims expressed in this article are solely those of the authors and do not necessarily represent those of their affiliated organizations, or those of the publisher, the editors and the reviewers. Any product that may be evaluated in this article, or claim that may be made by its manufacturer, is not guaranteed or endorsed by the publisher.
