# Supplementary material for: Growth Performance, Blood Biochemical Indices, Rumen Bacterial Community, and Carcass Characteristics in Goats Fed Anthocyanin-Rich Black Cane Silage
Source: Front Vet Sci. 2022 Apr 25;9:880838. doi: 10.3389/fvets.2022.880838 (PMC9101464; doi:10.3389/fvets.2022.880838)
Supplement: Supplementary file 1 [file Table_1.DOCX]

Supplementary Material

**TABLE S1 Chemical composition and fermentation characteristic of Napier grass silage (NS) and anthocyanin-rich black cane (AS)**

| **Item** | **Experimental diet** | |
| --- | --- | --- |
|  | **NS** | **AS** |
| Chemical composition, DM basis | | |
| Dry matter, % | 15.5 | 15.4 |
| Crude protein, % | 6.6 | 6.4 |
| Neutral detergent fiber, % | 52.5 | 51.1 |
| Acid detergent fiber, % | 31.8 | 31.6 |
| Acid detergent lignin, % | 6.7 | 3.9 |
| Hemicellulose, % | 20.7 | 19.5 |
| Cellulose, % | 25.1 | 27.6 |
| Ash, % | 11.5 | 11.4 |
| Fermentation characteristic, DM basis | | |
| pH value | 4.76 | 4.74 |
| Lactic acid, % | 3.01 | 3.48 |
| Acetic acid, % | 2.27 | 2.65 |
| Propionic acid, % | 0.0 | 0.0 |
| Butyric acid, % | 0.0 | 0.0 |
| Ammonia nitrogen, % | 0.04 | 0.03 |
